# Regulatory Control and the Costs and Benefits of Biochemical Noise

**DOI:** 10.1371/journal.pcbi.1000125

**Published:** 2008-08-15

**Authors:** Sorin Tănase-Nicola, Pieter Rein ten Wolde

**Affiliations:** Foundation for Fundamental Research on Matter (FOM) Institute for Atomic and Molecular Physics, Amsterdam, The Netherlands; Broad Institute of MIT and Harvard, United States of America

## Abstract

Experiments in recent years have vividly demonstrated that gene expression can be highly stochastic. How protein concentration fluctuations affect the growth rate of a population of cells is, however, a wide-open question. We present a mathematical model that makes it possible to quantify the effect of protein concentration fluctuations on the growth rate of a population of genetically identical cells. The model predicts that the population's growth rate depends on how the growth rate of a single cell varies with protein concentration, the variance of the protein concentration fluctuations, and the correlation time of these fluctuations. The model also predicts that when the average concentration of a protein is close to the value that maximizes the growth rate, fluctuations in its concentration always reduce the growth rate. However, when the average protein concentration deviates sufficiently from the optimal level, fluctuations can enhance the growth rate of the population, even when the growth rate of a cell depends linearly on the protein concentration. The model also shows that the ensemble or population average of a quantity, such as the average protein expression level or its variance, is in general not equal to its time average as obtained from tracing a single cell and its descendants. We apply our model to perform a cost-benefit analysis of gene regulatory control. Our analysis predicts that the optimal expression level of a gene regulatory protein is determined by the trade-off between the cost of synthesizing the regulatory protein and the benefit of minimizing the fluctuations in the expression of its target gene. We discuss possible experiments that could test our predictions.

## Author Summary

Biochemical networks, consisting of biomolecules such as proteins and DNA that chemically and physically interact with one another, are the processing devices of life. Metabolic networks allow living cells to process food, while signal transduction pathways and gene regulatory networks allow living cells to process information. Experiments in recent years have demonstrated that these networks are often very “noisy”: the protein concentrations often fluctuate strongly. However, how this “biochemical noise” affects the growth rate or fitness of an organism is poorly understood. We present here a mathematical model that makes it possible to predict quantitatively how protein concentration fluctuations affect the growth rate of a cell population. The model predicts that fluctuations reduce the growth rate when evolution has tuned the average protein concentration to the level that maximizes the growth rate; however, when the average concentration deviates sufficiently from the optimal one, fluctuations can actually enhance the growth rate. Our analysis also predicts that the optimal design of a regulatory network is determined by the trade-off between the cost of synthesizing the proteins that constitute the regulatory network and the benefit of reducing the fluctuations in the network that it controls. Our predictions can be tested in wild-type and synthetic networks.

## Introduction

Cells continually have to respond and adapt to a changing environment. One important strategy to cope with a fluctuating environment is to sense the changes in the environment and respond appropriately, for example by switching phenotype or behavior. Arguably the most studied and best characterized example is the *lac* system, where the LacI repressor measures the concentration of lactose and regulates the expression level of the metabolic enzyme that is needed to consume lactose. In this strategy of responsive switching, it is critical that cells can accurately sense and respond to the changes in the environment [1]. However, both the detection and the response are controlled by biochemical networks, which can be highly stochastic [2]–[11]. One might expect that noise is detrimental, since it can drive cells away from the optimal response curve—the optimal enzyme concentration as a function of the lactose concentration [12]. On the other hand, both reducing noise and creating a regulatory network that allows cells to respond optimally can be energetically costly [12], which would tend to reduce the fitness of the organism [13]. In this paper, we present a model that makes it possible to quantify the effects of biochemical noise on the growth rate of a population of cells that respond via the mechanism of responsive switching. We then use this model to perform a cost-benefit analysis of gene regulatory control, using cost and benefit functions that have been measured experimentally [12]. This analysis, which complements recent work by Kalisky and coworkers [14], predicts that gene regulatory proteins exhibit an optimum expression level, which is determined by the trade-off between the cost of synthesizing the regulatory protein and the benefit of reducing the fluctuations in its target gene.

It has long been recognized that organisms in a clonal population can exhibit a large variation of phenotypes. Within highly inbred lines, for instance, phenotypic variation can still be detected [15]. More recently, experiments have vividly demonstrated that gene expression in uni- and multicellular organisms fluctuates strongly [2]–[11]. The fact that fluctuations are not selected out, suggests that the optimal fitness requires a certain amount of biochemical noise. However, how the growth rate of a population depends upon biochemical noise is still poorly understood. In a constant environment, stabilizing selection favors a genotype that leads to a narrow phenotype distribution centered around the optimal phenotype in that environment [13],[16]. However, cells do not live in a constant environment, but rather in one that fluctuates. While one strategy to cope with environmental fluctuations is to detect and respond to them (responsive switching), an alternative one is to create diversity in the population. This can be achieved via the mechanism of stochastic switching [17]–[20], whereby members of the population randomly flip between different phenotypes due to biochemical noise. This strategy is particularly efficient when the time scales of the environmental fluctuations are either very long, such that the investments of constructing an energetically expensive response machinery do not pay off [20], or very short, i.e. shorter than the time it takes for the population to respond to them [18],[19]. Many examples of this strategy exist in nature [21],[22], and this strategy has recently been studied in much theoretical detail [17]–[20]. However, the dominant strategy for coping with changes in pH, temperature, the food supply or the presence of various toxic chemicals appears to be responsive switching. In this paper, we present a generic model that makes it possible to quantify the effect of biochemical noise on the growth rate of a clonal population of cells that use this mechanism to respond quickly to changes in the environment.

Our model integrates a description of how the internal dynamics of the composition of a cell affects the growth rate of that cell with a description of how the growth rates of the individual cells collectively determine the growth rate of the population. This allows us to address a number of fundamental questions: (a) How does the growth rate of the population depend upon the growth rate of a single cell as a function of its protein expression levels? (b) How does the population's growth rate depend upon the variance and the correlation time of these fluctuations? Our model predicts that an important parameter that controls the effect of biochemical noise is the correlation time of the fluctuations: only when the correlation time is long compared to the cell cycle time, does biochemical noise affect the growth rate of the population. Interestingly, recent experiments on *E. coli* [8] and human cells [11] have revealed that the correlation times of protein concentration fluctuations can be on the order of the cell cycle time, or even longer. Our analysis thus predicts that biochemical noise can significantly effect the growth rate of a population of cells. Moreover, our model predicts that fluctuations can both enhance and reduce the population's growth rate. When the average expression level of a protein is close to its optimum, fluctuations in its concentration will reduce the population's growth rate. However, when it is sufficiently far from its optimal level, fluctuations can actually enhance the growth rate of the population. This effect arises at the population level and is a consequence of the fact that cells that happen to growth faster due to noise, become overrepresented in the population.

Our analysis highlights the difference between ensemble averages and time averages [23]. The ensemble or population average of a quantity such as protein noise is defined as the average of that quantity over the cells in the population at a given moment in time; when a large population exhibits stationary growth, this average does not change with time. The time average of a quantity is defined as the average of that quantity in a single cell and its descendants over time. The time average is a property of the intracellular biochemical network: its value only depends upon the dynamics of the protein concentrations. In contrast, in experiments often the ensemble average is measured [3]–[6]. Our analysis elucidates that the ensemble average of a quantitylike the average expression level depends not only upon the dynamical properties of the network, but also on whether fluctuations of this quantity are coupledto the growth rate; if this is the case, then the ensemble average may differ significantly from the time average.

The model also allows us to perform a cost-benefit analysis of regulatory control. Recently, Dekel and Alon performed a series of experiments that strongly suggest that protein expression is the result of a cost-benefit optimization problem [12]. They showed that the expression level of the *lac* operon is determined by the trade off between the cost of synthesizing the metabolic enzyme LacZ and the benefit this enzyme confers in enabling the consumption of the sugar lactose. In particular, they developed a cost-benefit analysis that allowed them to successfully predict the optimal *average* expression level of the operon as a function of the lactose concentration. However, this analysis does not answer the question how the growth rate depends upon the fluctuations in the expression level of the metabolic enzyme, nor does it answer the question what determines the optimal average expression level of the gene regulatory protein that regulates the expression level of the metabolic enzyme.

While the cost function of synthesizing a gene regulatory protein is probably similar to that of producing a metabolic enzyme, their benefit functions are fundamentally different. The benefit of producing a metabolic enzyme is that it allows the uptake of the sugar by the metabolic network. In contrast, the benefit of synthesizing a regulatory protein is indirect and is derived from that of the metabolic enzyme; synthesizing a regulatory protein can be beneficial because it allows the cell to adjust the expression level of the metabolic enzyme to its optimum in response to a changing sugar concentration. However, a given optimal expression level of the metabolic enzyme as a function of the sugar concentration, does not uniquely determine the optimal expression level of the regulatory protein. A given optimal response function of the enzyme expression level as a function of the sugar concentration, can be obtained by different combinations of parameters such as the binding affinity of the inducer to the regulatory protein, the binding strength of the regulatory protein to the DNA, the degree to which these molecules bind cooperatively with each other, as well as the total concentration of the regulatory protein. What determines the optimal combination of these parameters that all can yield the same response curve of the enzyme expression level as a function of sugar concentration?

We conjecture that the benefit function of the regulatory protein is determined by the fluctuations in the expression level of its target, the metabolic enzyme, although other factors such as the response time could play a role as well. As we will show, when the average expression level of the metabolic enzyme is close to its optimum, fluctuations will tend to reduce the population's growth rate. Different gene regulatory networks can yield the same average response function, but can have markedly different noise properties. In particular, our analysis predicts that the inducer, e.g., sugar, should bind the gene regulatory protein strongly in order to reduce the fluctuations in the enzyme concentration. Moreover, it predicts that higher expression levels of the regulatory protein lower the noise in the expression level of the metabolic enzyme. We therefore predict that the optimal expression level of a regulatory protein is determined by the interplay between the cost of making the regulatory protein and the benefit of reducing the fluctuations in the target gene. Recently, a similar idea has independently been proposed by Kalisky, Dekel, and Alon [14]. Using as inputs the cost and benefit functions as measured by Dekel and Alon [12], our model predicts that the optimal expression level of the *lac* repressor should be on the order of 10–50 copies, which is remarkably close to the level found *in vivo* [24].

## Results

### Growth Rate

In order to describe the effects of biochemical noise on the growth rate of a population of cells, we have to develop a model that describes how (a) the internal dynamics of a cell affects the growth rate of that cell and (b) how the latter affects the growth rate of the population of cells. We now first discuss the latter.

#### The growth rates of single cells and the growth rate of the population

In order to quantify the growth rate of a cell, we have to define a parameter that monitors the progress along the cell cycle. This parameter, *Z*, could be the amount of replicated DNA, the length of the cell, or a combination of these parameters. It has a value *Z* = *Z*_i_ at the beginning of the cell cycle and a value *Z* = *Z*_f_ at the end of the cell cycle. The value of the ‘cell cycle coordinate’ *Z* thus exhibits an oscillatory sawtooth pattern as a function of time. Its role is analogous to that of a reaction coordinate in chemical kinetics, which measures the progress of a chemical reaction and serves to define the chemical rate constant. In our case, *Z* serves to quantify the instantaneous growth rate, λ, of each cell in the population:
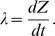
(1)


The growth rate λ depends upon the composition of the cell. This is determined by the expression level of ribosomal proteins, which are needed to make new proteins, and the expression levels of metabolic enzymes and other non-ribosomal proteins, which are required to produce the building blocks for protein synthesis and cell growth [25]. We denote the concentrations of these different proteins by{*X*_1_,*X*_2_,…,*X_n_*_−1_,*X_n_*}≡**X**. The growth rate λ is thus a function of **X**: *λ*≡*λ*(**X**). Together with the cell cycle coordinate *Z*, **X** specifies the state of each cell in the population.

To determine the growth rate of a population of cells, a key quantity is the probability density *P*(*Z*,**X**,*t*) to find a cell with a certain state *Z*, **X**, inside the population. The evolution of this probability density can be expressed in operatorial form as
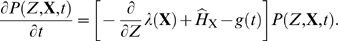
(2)


The first term on the right-hand side describes the evolution of *P*(*Z*,**X**,*t*) due to the deterministic evolution of *Z* (see Equation 1); it corresponds to a Fokker–Planck operator [26] in the limit of zero noise. The operator *Ĥ*_X_ is the Fokker-Planck operator encoding the evolution of *P*(*Z*,**X**,*t*) resulting from the noisy dynamics of the composition **X**. The last term describes the effect of cell division on the probability density *P*(*Z*,**X**,*t*). Indeed, the cell division at *Z*_f_ amounts to a “dilution” of the probability of finding cells with intermediate *Z* values. The steady-state probability distribution function, *P*_s_(*Z*,**X**,*t*), satisfies the equation
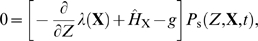
(3)with the boundary condition
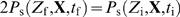
(4)


This condition formalizes the observation that upon cell division a cell at the end of the cell cycle gives birth to two newborns. Importantly, *g* is the growth rate of the population of cells in steady state. In this “stationary state,” the number of cells in the population grows exponentially, but the fraction of cells *P*(*Z*,*X*) with internal states *Z*, *X* has converged to a time-invariant quantity. At each moment in time, there is a constant fraction of cells ready to undergo cell division; the number of cells undergoing cell division thus grows exponentially with time, but remains proportional to the population size, with the proportionality factor given by the growth rate *g*.

#### Protein concentration fluctuations

The above model is a generic model of the cell cycle. To make further progress, we have to specify the dynamics of **X**. The copy number of a protein will increase as the cell grows, and will (on average) be divided in half when the cell divides. The copy number will thus exhibit an oscillatory temporal profile. The volume of the cell will show similar oscillatory dynamics. These oscillations will tend to cancel each other in their ratio, the *concentration* of the protein. We make the simplifying assumption that the concentration of each species fluctuates around a *constant* steady-state level during the cell cycle, and that the amplitude of these fluctuations is small. It allows us to linearize the interactions between the different species at steady state, and to use the linear-noise approximation [27]; a comparison with a description based on the chemical master equation has shown that this approximation is surprisingly accurate, even when the copy numbers are as low as ten [28],[29]. It yields the following set of chemical Langevin equations:
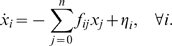
(5)


Here, *x_i_* = *X_i_*−*X*_s,*i*_ is the deviation of the concentration *X_i_* of species *i* away from its steady-state value *X*_s,*i*_, and *f_ij_* corresponds to the coupling between species *i* and *j*. The term *ξ_i_* describes the noise in *x_i_* that arises from the stochastic character of the chemical reactions. We model it as Gaussian white noise, with zero mean and variance determined by the concentrations of the species at steady state. In Equation 2, the relevant probability density now becomes *P*(*Z*,**x**,*t*) and the operator that describes the evolution of *P*(*Z*,**x**,*t*) due to the Langevin dynamics of **x**, becomes *Ĥ* *_x_* (see *Methods*).

#### The growth rate of a single cell as a function of protein concentration

If the composition of the cells would not fluctuate in time, then the evolution of the cell cycle parameter *Z* would be deterministic. The growth rate *λ*(**X**) of each cell would then be constant in time, *λ*(**X**) = *λ*_0_, and proportional to the growth rate of the population, *λ*_0_∼*g*. In the presence of biochemical noise, the growth rate not only depends upon the average protein levels, **X**, but also upon the fluctuations around the average, **x**, which lead to variations in the growth rate. It is conceivable that the growth machinery responds slowly to fluctuations in the composition in the cell; the growth rate would then “average” over fluctuations in the composition over some characteristic time scale *τ*: *λ* = *λ*(**X** *_s_*,**x̅** *^τ^*), where the bar with the superscript *τ* indicates that the fluctuations in **x** are averaged over a time τ. However, experiments have revealed that protein concentrations fluctuate fairly slowly: for *E. coli*, the correlation time is on the order of 45 min, which is on the order of the cell cycle time [8]. We argue that since the protein concentrations relax slowly, it is reasonable to assume that the instantaneous growth rate depends upon the instantaneous composition of the cell. We therefore conjecture that the growth rate is given by *λ* = *λ*(**X** *_s_*,**x**).

To obtain the growth rate *λ*(**X** *_s_*,**x**), we expand it around the steady state **X** *_s_* to second order in **x**

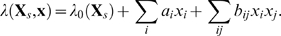
(6)


The equation for the stead-state probability density *P*_s_(*Z*,**x**,*t*), Equation 3, can now be solved by making a multidimensional Gaussian Ansatz for *P*_s_(*Z*,**x**,*t*)
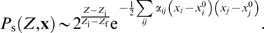
(7)


From now on we shall rescale the time and the *Z* coordinate such that *Z*_f_−*Z*_i_ = log(2). In order to understand why such a transformation is useful, it should be noted that in the absence of protein concentration fluctuations, each cell in the population needs a constant time between birth and division *T*_cycle_ = (*Z*_f_−*Z*_i_)/*λ*_0_. At the population level, *T*_cycle_ is also the time it takes for the population to double in size, such that the growth rate of the population is *g* = log(2)/*T*_cycle_. Clearly, in the zero fluctuation limit, the growth rate of the population of cells equals the growth rate of each single in the population: *g* = *λ*_0_. In the presence of protein concentration fluctuations, however, the cell cycle times of the individual cells will fluctuate, such that even a population of cells that are initially perfectly synchronized will eventually converge towards a steady-state distribution as given by Equation 7.

### Time Averages Do Not Always Equal Ensemble Averages

Our model shows that the “time average” of a quantity such as the average protein expression level or the noise in gene expression, is, in general, not equal to its “ensemble average” [23]. The time average of a quantity *X*, *X̅*, is defined as the temporal average of *X* along one “line of descent”:
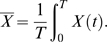
(8)


Here, *X*(*t*) can be obtained by monitoring *X* as a function of time in a given cell, whereby upon cell division one follows a randomly chosen descendant. The integration time *T* should be much longer than the correlation time of the fluctuations in *X*. To obtain better statistics, one could average over different trajectories *X*(*t*) in a population, but each such path has to have a different ancestor (the first cell on the path). The ensemble average of the quantity *X*, 〈*X*〉, is defined as the average of *X* across the population of cells:
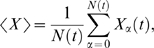
(9)where *N*(*t*) is the number of cells in the population at time *t* and *X_α_*(*t*) is the magnitude of *X* in cell *α* at time *t*; when the growing population is in the stationary state and *P*(*Z*,**X**,*t*) is time invariant, this ensemble average does not change with time. To illustrate the difference between the two kinds of averages, let's consider the fluctuations in the composition **X**. To the extent that protein concentration fluctuations are described by the chemical Langevin equation (Equation 5), the distribution of the concentrations **X** as obtained by following the time traces of *X_i_* in a given cell and its descendants, is given by a Gaussian that is centered at **X̅** = **X**_s_. In contrast, the distribution of **X** over different cells in a population at a given moment in time is also a Gaussian, but now the Gaussian is centered at 〈**X**〉 = **X**_s_+**x** ^0^, where **x** ^0^ may deviate from zero. Moreover, not only the mean, but also the variance of the two distributions will, in general, differ, as we will show now.

### Biochemical Noise Can Both Reduce and Enhance the Population's Growth Rate

In order to understand the non-trivial effects of biochemical noise on the growth rate of a population of cells, it is instructive to consider a simple example. Let's consider a single metabolic enzyme X, and assume that the temporal dynamics of its concentration is given by
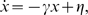
(10)where *x* is the deviation of the enzyme concentration *X* away from its steady-state value, *X*_s_, γ^−1^ is the response time, which is typically on the order of the cell cycle time, and η is a Gaussian white noise term, of zero mean and strength 2*D*. The time average of the variance of the fluctuations in the concentration of X as obtained from the time trace of *X* of a given cell and its descendants, is 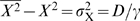.

We assume that over the concentration range of interest, the growth rate of a given cell as a function of the expression level of X can be written as
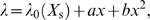
(11)where *λ*_0_(*X*_s_) is the growth rate of the cell when the enzyme concentration equals *X*_s_. The growth rate of the population of cells is then given by (see *Methods*)
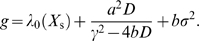
(12)


Here, *σ* ^2^ is the variance of the fluctuations in *X* within the population of cells at a given time: σ^2^ = 〈*X* ^2^〉−〈*X*〉^2^. This ensemble or population average is given by
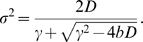
(13)


The ensemble average *σ* ^2^ can be written in terms of the time average of the variance 
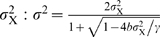
. Clearly, if the growth rate is non-linear in *X*, i.e., if *b*≠0, the ensemble average of the variance in *X* does not equal its time average. Importantly, the time average of the protein noise, 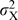, is a characteristic of the stochastic properties of the underlying biochemical network. However, the protein noise is often measured as an ensemble or population average [3]–[6]. Our results show that if one is interested in the noise properties of the underlying network, one should compute the protein noise by combining sequential noise traces of cells through lines of descent [30] when the expression of the fluorescent protein used to measure the noise affects the growth rate significantly (such that *b* is much smaller than zero).

**Figure 1:**
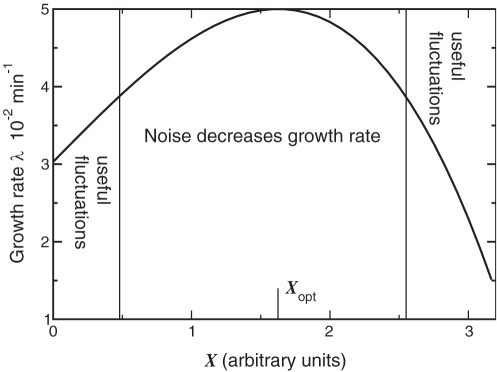
A Sketch of the Instantaneous Growth Rate λ of a Single Cell as a Function of the Concentration *X* of Component X. If the average expression level *X*_s_ is close to the optimal expression level *X*_opt_, biochemical noise will always decrease the growth rate. If, however, the average expression level deviates sufficiently from the optimal expression level (i.e. if *ax*>*bx* ^2^ in Equation 11), then fluctuations can enhance the growth rate of the population, even when the growth rate λ of a single cell is linear in *X*, i.e. if *b* = 0. The reason is that fast growing cells dominate the population.

Let us now consider the scenario in which the average expression level of the enzyme is such that the growth rate is maximal: *X*_s_ = *X*_opt_ (see Figure 1). In this case, *a* is zero, and *b* = ∂^2^ *λ*/∂*X* ^2^<0. The growth rate of the population is then *g* = *λ*_0_(*X*_s_)+*bσ* ^2^. Since *b* is negative, *g*<*λ*_0_. Hence, when the composition is close to its optimum, biochemical noise always tends to reduce the overall growth of the population.

If the average expression level *X*_s_ deviates significantly from the optimal expression level *X*_opt_, the situation is qualitatively different (see Figure 1). Sufficiently far away from the optimum, the curvature can be ignored (*b* = 0), and the growth rate is given by 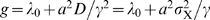. In this regime noise always increases the growth rate, irrespective of the sign of *a*, and even though at the *single cell* level the growth rate *λ* is linear in *X*. The reason is that cells that happen to have a composition that is closer to the optimum, will grow faster and therefore divide earlier; moreover, the daughter cells will inherit the composition from their mother, and will thus also grow faster than the steady-state value, and so on. As a consequence, cells with a higher growth rate become overrepresented in the population, which can be verified by noting that the mean of *x* in the population of cells is now shifted from zero to 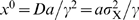. This mechanism, whereby the cells that grow faster due to a fluctuation in their protein composition generate more off-spring, increases the overall growth rate of the population. The increase in the growth rate due to noise, 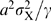, depends upon how strongly the growth rate changes with *X*, which is given by the slope *a*, and on the magnitude of the concentration fluctuations in each cell, given by 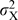. Importantly, it also depends upon the relaxation time of the fluctuations, given by γ^−1^. If the response time is much faster than the cell cycle time, then on the relevant time scale of the cell cycle, the concentrations in all the cells will be the same and no benefit from the noise can be gained. However, both in prokaryotic [8] and eukaryotic cells [11], correlation times of protein concentration fluctuations have been measured to be on the order of the cell cycle time or longer, meaning that they are potentially important. Please also note that a non-zero *x* ^0^ means that the time average of *X*, which is given by *X̅* = *X*_s_, is not equal to the ensemble average of *X*, which is given by 〈*X*〉 = *X*_s_+*x* ^0^.

Lastly, we note here that it is conceivable that the curvature *b* of the growth rate *λ* is locally positive. In this case, the solution to Equation 12 is only valid when *γ* ^2^>4*bD*. At the point where this condition is no longer satisfied, an interesting bifurcation can arise towards a state where the growth dynamics alone imposes a bimodal distribution of protein concentrations: in the population, cells with a high expression level then co-exist with cells with a low expression level.

### Fluctuating Environment

The analysis above describes how fluctuations in the composition can affect the growth rate of a population of cells in a constant environment. We now briefly discuss how fluctuations in the environment affect the population's growth rate. As before, we consider the scenario in which cells respond to changes in the environment via the mechanism of responsive switching: they thus sense the changes in the environment and respond appropriately.

If the environmental signals are described by the vector **S**, then the time varying environment can, in general, be decomposed as:
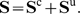
(14)


Here, **S** ^c^ denote the correlated fluctuations between the different cells, while **S** ^u^ corresponds to the fluctuations in the environmental signals that are uncorrelated from one cell to the next within the population.

The uncorrelated fluctuations in the external signals can be treated in the same spirit as the fluctuations in the internal signals. Their dynamics could be added to that of **x**:
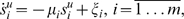
(15)

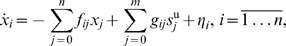
(16)where 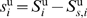, with 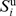 the part of the fluctuations of the external signal *i* that is uncorrelated between different cells, and *g_ij_* indicates how the internal dynamics of species *i* is coupled to the fluctuations in the external signal *j*. Since the fluctuations in **S** ^u^ couple to the fluctuations in the composition **X**, they could either reduce or enhance the growth rate of the population, depending on whether the composition **X** is close to its optimum or not, respectively.

The effect of the correlated fluctuations in the external signals, **S** ^c^, are much more difficult to treat analytically [18]. However, if these fluctuations occur on a time scale that is much longer than the time it takes for the internal dynamics x to relax towards a new steady state after an environmental change, the overall growth rate can be written as
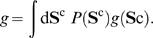
(17)This expression shows that the cells need to adapt to a given distribution of external signals.

We can make an estimate for the time it takes for the population to relax towards a new steady after a change in the environment has occurred. If prior to an environmental change, the cell cycle coordinate *Z* has reached steady state, meaning that *P*(*Z*) is uniform across the population of cells, then *P*(*Z*) does not have to relax towards a new steady state after the change in the environment. The distribution in the composition, *P*(*X*), however, does have to relax. If the relaxation time of the population is dominated by the slow dynamics of a single protein *X*, the relaxation rate is given by 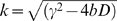. This shows that in the absence of fluctuations (*D* = 0) the relaxation rate is given by the rate of protein decay, *γ*, as one would expect. It also shows that when the growth rate of a cell is a concave function of *X* (*b*<0), fluctuations can actually enhance the relaxation rate; the reason is that cells that are closer to the new optimum will grow faster. This analysis shows that a conservative estimate for the validity of Equation 17 is that the environmental fluctuations should occur on time scales longer than the protein decay time *γ*.

### The Cost of Reducing Noise: Optimal Expression Levels of Gene Regulatory Proteins

In order to understand the design criteria that determine the magnitude of the fluctuations in the expression level of a given protein for cells that respond via responsive switching, we do not only have to understand how these fluctuations affect the growth rate, as discussed above, but also the indirect energetic cost of controlling these fluctuations. Both the magnitude of the concentration fluctuations and the cost of controlling these fluctuations are determined by the design of the network that regulates the expression level of the protein of interest. We will now show, using the *lac* system as an example, that the optimal design of the regulatory network is determined by the interplay between these two factors.

We use a simple model of the *lac* system in the absence of glucose but in the presence of lactose. The inducer lactose (ligand L) binds the lac repressor (transcription factor TF); upon binding, the transcription factor dissociates from the operator and the enzyme, LacZ in this case, is expressed. We assume that both the binding of ligand to the transcription factor and the binding of the latter to the operator are fast such that they can be integrated out. The dynamics of the regulatory protein and the metabolic enzyme is then specified as:
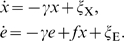
(18)


Here, *x* denotes the deviation away from the total steady-state TF concentration, denoted by *X*_s_, *e* denotes the deviation away from the steady-state concentration of the enzyme, *E*_s_, *γ* is the degradation rate of both proteins, and *ξ*_X_ and *ξ*_E_ model the (Gaussian white) noise in their expression. The factor *f* is the differential gain that describes the change in the protein production rate (expression rate) *k*_E_(*X*) due to a change in the concentration of the transcription factor: *f* = ∂*k*_E_(*X*)/∂*X*. In this expression we integrate the contributions of TF-ligand binding, TF-operator binding, and the dynamics of mRNA. The fluctuations in *e* have an intrinsic source, modeled by *ξ_e_*, and an extrinsic one that arises from the fluctuations in *x*. Since the expression level of the enzyme is much higher than that of the gene regulatory protein, the dominant source of noise in *e* is the extrinsic one, arising from fluctuations in the TF concentration. In what follows, we therefore ignore the intrinsic contribution *ξ*_E_.

To make further progress, we need to know how the growth rate of each cell, *λ*, depends upon the expression level of the enzyme and that of the transcription factor. Recently, Dekel and Alon [12] performed a series of experiments that allowed them to measure both the cost and the benefit of producing the metabolic enzyme LacZ. By using an artificial inducer, they varied the expression level of LacZ in the absence of its substrate lactose, and measured the effect on the growth rate. The inducer induces the production of LacZ, but no benefit is gained, since the lactose is absent and the inducer is not metabolized. This set of experiments thus allowed them to determine the cost of synthesizing the LacZ protein. In a separate set of experiments they measured how the growth rate changes with the lactose concentration, when the expression level is kept constant (due to a saturating amount of the inducer). This set of experiments gave them an (indirect) estimate of the benefit function. By assuming that the optimal expression level is given by the level that maximizes the benefit minus the cost, the measured cost and benefit functions could be used to predict the optimal LacZ expression level as a function of lactose concentration.

Following Dekel and Alon [12], we write the change in the growth rate of a single cell, Δ*λ* = *λ*−*λ*_0_, due to the production of the gene regulatory protein and the metabolic enzyme relative to the growth rate in the absence of these proteins, *λ*_0_, as:
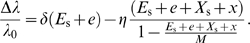
(19)


The first term on the right-hand side encodes the gain in the growth rate due to the metabolic activity of the enzyme; importantly, *δ*≡*δ*(*L*) is a function of the lactose concentration *L* (see Equation 26 below). The second term, with η being a constant, quantifies the cost of producing the enzyme and the regulatory protein; the factor *M* is the maximal capacity for producing non-essential proteins [12]. Note that we assume that the costs of producing one enzyme molecule and one gene regulatory protein molecule are the same.

As discussed in the introduction, a given average optimal expression curve of *E* as a function of sugar concentration, *E*_opt_(*L*), can be obtained by different expression levels of *X*. A mean-field analysis, which ignores the effect of fluctuations in *E* and *X*, would predict that the optimum expression level of *X* is close to zero, since that minimizes the cost of producing the regulatory protein. We therefore assume that the steady-state enzyme expression level, *E*_s_, is given by that level 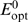 that maximizes Δ*λ* with respect to *E* at *X* = 0. The steady-state enzyme expression level is thus given by
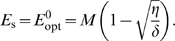
(20)


This expression is, in fact, the principal result of the cost-benefit analysis of the optimal enzyme expression level of Dekel and Alon [12]. The expression, with *δ* being a function of the lactose concentration (see Equation 26 below), gives a remarkably good prediction for the enzyme expression level as a function of the lactose concentration [12]. The prediction is shown in Figure 3 (panel C). We now address the question what is the optimal regulatory network—the optimal TF concentration *X*_s_, the optimal TF-L and TF-operator binding strengths—under the assumption that the steady-state enzyme expression level as a function of lactose concentration is fixed and given by Equation 20: 
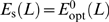
.

To obtain the growth rate at *E* = *E*_s_+*e* and *X* = *X*_s_+*x* (with finite *X*_s_), we expand the growth rate around 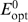 and *X* = 0, which yields the following expression for the relative growth rate (see *Methods*):
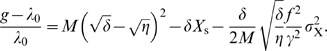
(21)


On the left-hand side of the above equation, *g* is the growth rate of the *population* of cells. The first two terms on the right-hand side give the deterministic, mean-field prediction that ignores the effect of fluctuations in *x* and *e*: in the absence of fluctuations, the growth rate of the *population* of cells, *g*, equals the growth rate of each single cell, *λ*_D_, which is given by 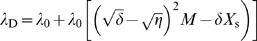 (see *Methods*). The last term of Equation 21 describes the effect of fluctuations on the growth rate. The second term on the right hand side shows that at the mean-field level, there is indeed a pressure to minimize the production of the regulatory protein X; this is associated with minimizing the cost of producing the regulatory protein. The third term on the right hand side shows, however, that there is also a pressure to minimize the fluctuations in X, given by 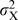. Its origin is that fluctuations in the gene regulatory protein *X* lead to fluctuations in *E*, and since the mean expression level of *E* is assumed to be at its optimum, these fluctuations tend to lower the growth rate. Importantly, the magnitude of the fluctuations in *X* and hence *E* decreases as the average expression level of *X* increases. Clearly, while the cost of producing *X* tends to lower the optimal expression level of *X*, the benefit of reducing the fluctuations in *E* tends to increase the optimal expression level of *X*. The optimal expression level of *X* is determined by the balance between these two opposing factors. A similar conclusion was recently independently reached by Kalisky et al. [14].

To demonstrate this explicitly, we will study in more detail the last two terms in Equation 21, which describe the contribution of the transcription factor to the growth rate:
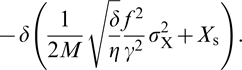
(22)


In our model, the steady-state enzyme concentration is given by *E*_s_ = *E*_opt_ = *k*_E_(*X*_s_,*L*)/γ, which means that the gain is given by
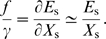
(23)


To make further progress, we have to assume a model for the fluctuations in *X*. If we assume that these fluctuations are Poissonian, then 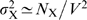 [3], where *V* is the volume and *N*_X_ is the copy number of *X*. Recent results show that while the fluctuations can be stronger than Poissonian due to, for example, bursts in gene expression, the linear scaling of 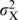 remains correct for many proteins in prokaryotes [31]. Finally, if we assume that *E*_s_∝*M*, the expression in Equation 22 is proportional to
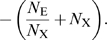
(24)


This expression shows a maximum as a function of *N*_X_. The position of this optimum—the copy number of X that maximizes the growth rate—is related to the copy number of E by
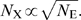
(25)


We therefore predict that the optimal TF copy number is linear in the square root of the copy number of the enzyme it regulates. This prediction could perhaps be tested by performing a statistical analysis of the expression levels of transcription factors and the expression levels of the target genes these transcription factors regulate. Such a statistical analysis could be performed in the spirit of that of [31], in which the authors studied the variation in the expression levels of 43 *Saccharomyces cerevisiae* proteins, in cells grown under 11 experimental conditions. Our analysis would predict that if one would measure the expression levels of transcription factors and their target genes in such an experiment, the two would be correlated according to Equation 25.

Dekel and Alon [12] measured the quantities *δ* and *η* used above (Equation 19) for the *lac* system:
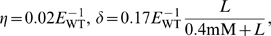
(26)(where *L* is measured in mM units). Here *E*_WT_ is the fully induced wild-type concentration of the enzyme, and we use *M* = 1.8*E*_WT_. As explained in the section *Fluctuating Environment* the growth rate in a slowly fluctuating environment can be obtained as an average over the different levels of the lactose in the environment. As we do not know the wild type distribution of sugar the bacterium experiences, we use either a uniform distribution over all possible lactose levels in the interval 0–6 mM or a non-uniform bimodal one that peaks at small and high lactose concentrations.

**Figure 2:**
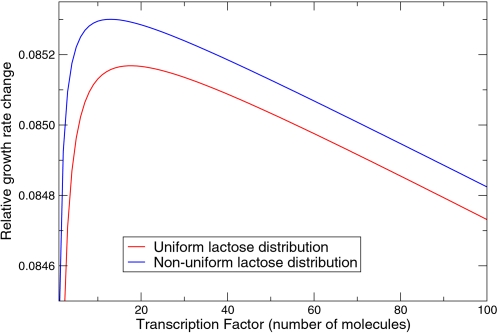
Relative Change in the Growth Rate as a Function of the Average Repressor Concentration. The growth rate is averaged over different lactose concentrations in the environment (see Equation 17), for two different lactose concentration distributions in the environment.

Figure 2 shows the optimal repressor expression level, for the two different lactose distributions in the environment. It is seen that the growth rate as a function of the copy number of the regulatory protein exhibits a broad optimum at around 10–50 molecules. Interestingly, this is in the biological range [24]. Even though our model of gene expression is rather simplified (we use, e.g., a constant amplification factor *f*), it appears that the prediction of our model is remarkably accurate. Interestingly, Kalisky et al. arrived at a similar prediction, even though their model differs in a number of ways from ours, as discussed in more detail in the *Discussion* section [14].

Equation 21 shows that the effect of the noise in *X*, 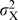, on the fluctuations in E, and hence on the growth rate, is determined not only by the decay rate *γ*, which controls the extent to which fluctuations in *X* and *E* lead to significant differences between cells in their composition on the time scale of the cell cycle, but also by the gain *f*, which determines the extent to which the fluctuations in *X* are amplified. As we will show now, the optimal TF-ligand binding curve and TF-operator binding curve is determined by the requirement that the gain *f* should be minimized as much as possible. Let's imagine that the binding of the ligand *L* to the repressor *X* is given by
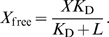
(27)


Here, *X* is the total TF concentration, *X*_free_ is the concentration of *X* that is not bound to the inducer, and *K*_D_ is the dissociation constant for ligand-TF binding. The unbound transcription factor represses the expression of *E* via the repression function *R*≡*R*(*X*_free_(*X*,*L*)), given by
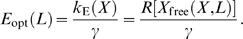
(28)


**Figure 3:**
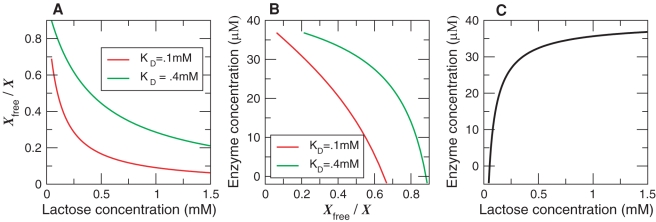
Different Regulatory Networks Can Yield the Same Optimal Enzyme Expression Level as a Function of Inducer Concentration. This is illustrated for two regulatory networks of the *lac* system, which differ in the dissociation constants of lactose-repressor binding and repressor-operator binding. Panels (A) and (B) show the response functions at two different stages of the *lac* regulatory network, while panel (C) shows the resulting optimal enzyme expression level as a function of lactose concentration. (A) The fraction of repressor that is not bound by lactose, *X*_free_/*X*, as a function of lactose concentration for two different lactose-repressor binding constants. (B) The corresponding response curves of the enzyme expression level as a function of the fraction of free repressor. The total expression level of repressor is chosen to correspond to the optimal growth rate (see Figure 2). (C) The resulting optimal enzyme expression level as a function of the lactose concentration, as predicted by Equation 20 [12].

We show these relations in Figure 3. It is important to note that the repression function *R*(*X*_free_(*X*,*L*)) is not necessary a simple Hill function; in the *lac* system this curve is known to be implemented with a complicated cooperative interaction and binding to multiple operator sites on DNA. Using Equation 23, 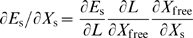 and Equation 27, we arrive at
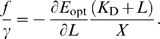
(29)


**Figure 4:**
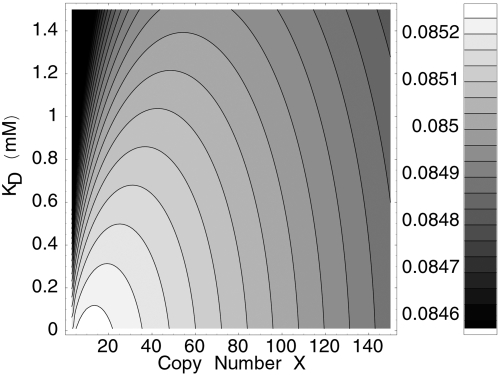
The Optimal Design of the *lac* Regulatory Network Is Determined by the *lac* Repressor Copy Number and the Repressor–Lactose Binding Constant. Contour plot of the growth rate as a function of the repressor copy number *X* and repressor-lactose binding constant *K*_D_. The weighting of the lactose levels is nonuniform. Lower binding constants allow for higher optimal growth rates at lower optimal expression levels for the repressor.

To minimize the gain *f*, and hence the effect of noise in *X* on the growth rate, *K*_D_ should be as small as possible, which corresponds to strong TF-L binding. Since the function *E*_opt_(*L*) is assumed to be fixed, strong TF-L binding also implies strong TF-operator binding. Hence, as long as TF-ligand binding and TF-operator binding can be integrated out, the best strategy would be strong TF-L and TF-operator binding. This is illustrated in Figure 4, which shows for the *lac* system the contour plot of the optimal growth rate in the plane (*X*,*K*_D_). The conclusion that TF-L and TF-operator binding should be strong is supported by the experimental observation that the dissociation constant for the binding of lac repressor to its primary operator site is in the nM range, while the binding of the inducer allolactose to the repressor is on the order of 0.1 µM [32].

## Discussion

The response machinery allows a living cell to adjust its composition to a changing environment. If the response machinery is fast and operates well, then in each environment the cell's composition is optimized such that the growth rate is maximized. Our analysis suggests that under these conditions, there is an evolutionary pressure to minimize the fluctuations in the composition. However, the response machinery cannot always optimally adjust the cell's composition. When there is a drastic change in the environment, for instance, the cell probably has to change its genotype so as to change its response machinery. Our analysis suggests that along such an “evolutionary trajectory” from a sub-optimal configuration of the response machinery to a new optimal one, fluctuations in the composition could be beneficial, because cells that happen to have a composition that is closer to the new optimum will grow more rapidly and thereby increase the overall growth rate of the population. Based on this observation we predict that the periods of fast evolution (for example when a population colonizes an entirely new environment) are correlated with a positive influence of fluctuations and thus an increased variability in the population. This idea is supported by the observation that the regulatory networks that control the response to environmental changes are in general noisier than the conserved cell machinery [31],[33].

It has been recognized before in a different context that phenotypic variance can be detrimental under stabilizing selection for the optimal genotype and advantageous far from this optimal genotype [13],[16]. Moreover, it has been suggested that phenotypic variance could be maintained if there is an “engineering” cost of minimizing fluctuations [13]. Our model, however, makes it possible to make a quantitative prediction on the effect of protein concentration fluctuations on the growth rate of a clonal population of cells. In particular, the model predicts that the effect of fluctuations in the concentration of a given protein X depends upon the following quantities (see Equation 12): (a) the growth rate of a single cell as a function of the expression level, *λ*(*X*) [12]; (b) the strength of the fluctuations in *X*, 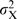; (c) the correlation time of the fluctuations in *X*, given by *γ*. All these quantities can be measured experimentally, which would allow for a quantitative test of our model. In this respect, it would be of particular interest to investigate one of the key ingredients of our model, which is how the growth rate of a single cell, *λ*, depends on the composition X. We have assumed that the growth rate depends upon the instantaneous composition, but it is conceivable that the growth rate responds to changes in the composition with a time lag; alternatively, it could depend upon the composition as averaged over some time scale *τ*: *λ* = *λ*(**X̅** *^τ^*).

Recently, Kalisky, Dekel and Alon [14] reported an analysis of the optimal design of the gene regulatory network that controls the expression of the *lac* operon, which complements ours. While we assume that the correlated fluctuations in the environment are slow, they also consider correlated fluctuations in the environment that are relatively fast to the response time; on the other hand, their analysis does not address the question of the optimal dissociation constants for inducer-TF and TF-operator binding. Our analyses also differ in the description of the extrinsic contribution to the noise in the expression of the *lac* operon, and in the estimate of the burst size of lac expression. More importantly, Kalisky et al. used a simpler model to describe the effect of biochemical noise on the growth rate of a population of cells. Our model integrates a description of the effect of noise on the growth rate of a single cell with a description of how the growth rates of the single cells collectively determine the growth rate of the population. In contrast, their model assumes that the growth rate of the population is given by the average of the growth rates of the individual cells. This approximation does not allow the model of Kalisky et al. to predict that the noise can also enhance the growth rate of the population. This is indeed an effect that arises at the population level; it is a consequence of the fact that cells that happen to grow faster will take over the population. Moreover, our work illustrates the importance of the correlation time of the protein concentration fluctuations. However, the present work agrees with that of Kalisky, Dekel, and Alon [14] in that we both find that the optimal concentration of a gene regulatory protein is determined by the interplay between the cost of synthesizing the regulatory protein and the benefit of reducing the fluctuations in the expression of its target gene. Even quantitatively, the predictions of our models for the optimal *lac* repressor concentration are fairly similar, although the model presented here would predict a slightly lower optimum concentration and a slightly smaller change in growth rate for deviations away from this optimum; this could be due to our conservative estimate of the burst size.

Our model predicts that if the expression level of the gene regulatory protein is varied by a factor 2 from its optimal value, the change in the growth rate would be on the order of 10^−4^. This change is sufficient to provide a selection pressure that is large enough in a typical bacterial population with an effective size larger than 10^6^ cells; indeed, as discussed in [34], relative growth rate changes as low as 10^−6^ are sufficient to balance the genetic drift in such a population. A change in the growth rate of 10^−4^ is thus large enough to provide a selection mechanism in a typical bacterial population for driving the transcription factor expression level to within a factor 2 from the predicted optimal level.

Another fundamental question we can address with our model is the relative efficiency, from the fluctuations point of view, of different modes of regulation (see *Methods*). For example, the cost-benefit function of Dekel and Alon implies that the cost grows with a linear combination of the total enzyme and transcription factor concentration, with positive coefficients [12]. As a consequence, regulatory networks with anticorrelated fluctuations of the enzyme and TF concentrations, which correspond to repressor based regulatory networks, will provide an advantage over those with correlated fluctuations, as for activator based regulatory networks. This result is consistent with the observation that simple organisms have more repressors than activators. Unlike alternative explanations for this observation based on the requirement for genotypic robustness with respect to mutational fluctuations [35],[36], our explanation does not require that the rate of environmental fluctuations is comparable to the slow relevant mutation rates.

In this paper, we have focused on the expression of a single protein. Yet, it is clear that the model presented in *Growth rate* could be used to study more complicated networks as well. In these networks, the propagation of noise [37]–[40] and hence the effect of noise on the growth rate, can be intricate, especially when there are (anti-) correlations between different sources of noise [28],[41]. The model could also be used in conjunction with partial-differential equation solvers to study non-linear networks, for which biochemical noise is expected to become even more important.

How could our predictions be tested experimentally? Ideally one would like to perform an experiment in which the *average* expression level of the metabolic enzyme is fixed, while the noise in the expression level is varied. Several strategies could be envisioned. First of all, one could vary the noise level by playing with the transcription and translation efficiencies [3],[38]. To make more direct contact with the predictions presented here, however, it would perhaps be more interesting to vary the expression level of the regulatory protein, while simultaneously varying the TF-operator binding strength such that the average expression level of the metabolic enzyme remains constant. Alternatively, one could vary the expression level of the regulatory protein, while simultaneously changing the concentration of an artificial inducer such that the enzyme concentration remains constant. For example, it is possible to increase the binding affinity of the *lac* repressor to the operator, and therefore the repression strength by a factor as high as 10, by either mutating the repressor LacI [42] or the operator sites [43]. Our analysis predicts that the growth rate as a function of the expression level of the regulatory protein exhibits a broad maximum as shown in Figure 2.

## Methods

### The Stationary Distribution ***P*** **_s_**(***Z***,x)

In this section we derive the solution (Equation 7) for the stationary probability distribution *P*_s_(*Z*,**x**). The equation satisfied by *P*(*Z*,**x**,*t*) for the case of linear Langevin dynamics is:
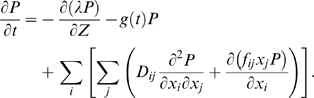
(30)


The three terms on the right hand side of Equation 30 describe, in order, the drift along the cell-cycle coordinate *Z*, the normalization of *P* due to the continuous birth of new cells in the population, and the Fokker-Plank operator describing the internal dynamics of the composition of the individual cells [26],[44]. The noise strength *D_ij_* is given by 〈*η_i_η_j_*〉 = 2*D_ij_*, where 〈*η_i_η_j_*〉 are the cross-correlations in the Gaussian white noise of *X_i_* and *X_j_* [28]. The stationary solution satisfies the equation:
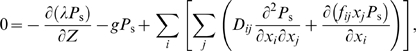
(31)with the boundary condition 2*P*_s_(*Z*_f_,*x*) = *P*_s_(*Z*_i_,*x*).

The instantaneous growth rate is given by:
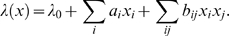
(32)For the stationary distribution we make the Ansatz
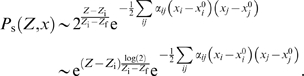
(33)Using the scaling *Z*_i_−*Z*_f_ = log(2) we obtain
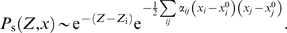
(34)If we insert this Ansatz into Equation 31, we obtain
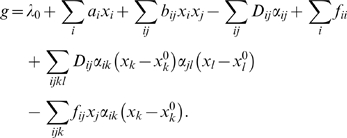
(35)


For this multidimensional polynomial equation to be satisfied for all the values of **x**, all the coefficients must be zero. Therefore the growth rate is given by:
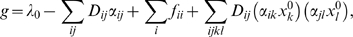
(36)where the constants α and **x** ^0^ must satisfy the set of 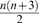 equations:
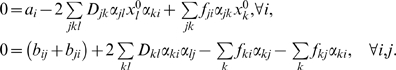
(37)


We can read from the Equations 37 that negative curvatures of the instantaneous advancement rate (*b_i_*<0) concentrate the Gaussian stationary distribution *P_s_*(*Z*,*x*) (induce larger *α*'s), while non-zero values for *a_i_* displace the averages 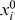 of the Gaussian stationary distribution *P_s_*(*Z*,*x*) such that 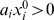.

### Growth Rate Controlled by a Single Enzyme

We derive here Equation 12. As discussed in the text, we model the dynamics of enzyme X via the linearized Langevin dynamics,
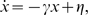
(38)while we assume that the growth rate of a single cell as a function of the expression level of X can be written as
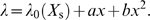
(39)We must solve the equation
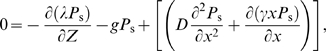
(40)where we choose *D* such that the strength of the biochemical noise *η* is 2*D* [44]. To obtain the stationary distribution, we make the Ansatz
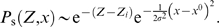
(41)


If we insert this into Equation 40, we find that we have to solve the equations
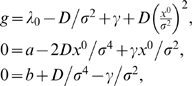
(42)from which we obtain the solution
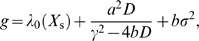
(43)

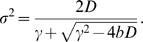
(44)


### Cost-Benefit Analysis of Gene Regulation

We now present the derivation and the approximations leading to Equation 21. A mean-field analysis of the cost-benefit function of Dekel and Alon [12], Equation 19, predicts that the maximum growth rate occurs at 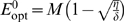 and *X* = 0. We are interested in the growth rate of a cell in which the average enzyme concentration is 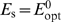, while the average transcription factor concentration, *X*_s_, is finite. Since the average transcription factor concentration, *X*_s_, is nevertheless small, it is reasonable to assume that the growth rate of a cell with *E* = *E*_s_+*e* and *X* = *X*_s_+*x* can be obtained by Taylor expanding the growth rate given by Equation 19 around the deterministic prediction, 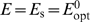, *X* = 0. This yields
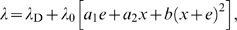
(45)where
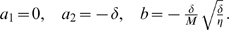
(46)


Here, *λ*_0_ is the growth rate of each single cell when the gene regulatory protein and the enzyme are not expressed [45]. The rate *λ*_D_ is the “deterministic” growth rate, thus the growth rate when the regulatory protein and the enzyme are expressed, but fluctuations are not taken into account. It is given by:
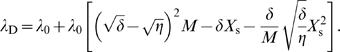
(47)Remark that at zero *X*_s_ we have:
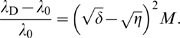
(48)


Equations 36 and 37 can now be solved using Equations 45–47 to obtain the growth rate that takes into account the noise. This leads to the following expression for the growth rate:
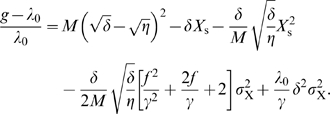
(49)


In deriving Equation 49 we also use the fact that the transcription factor concentration is much smaller than the typical enzyme concentration, yielding 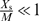. We also use the inequalities 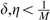 [45] and the Poissonian nature of the noise in the transcription factor: 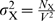. Equation 49 can be further simplified by keeping in our approximation only the terms of order one or larger in the small ratio 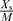. Please note that in the absence of fluctuations, the above equation reduces to *g* = *λ*_D_: the growth rate of the population of cells, *g*, then equals the growth rate of each single cell, *λ*_D_.

The last term in Equation 49 is positive, and, interestingly, *promotes* fluctuations in *X*. It comes from the finite derivative at *X* = 0, as explained in *Biochemical noise can both reduce and enhance the population's growth rate*. However,
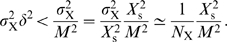
(50)


Therefore, the last term in Equation 49 is negligible at our level of approximation.

We also have
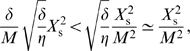
(51)while
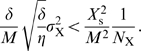
(52)We can therefore simplify Equation 49 to the form
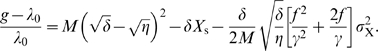
(53)


Around the steady state 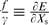, and we thus also have 
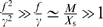
, such that we can simplify Equation 53 further. Nevertheless, it is important to remark that positive regulation (*f*>0) increases the detrimental effect of fluctuations in the concentration of the gene regulatory protein. Hence, at this level the cost of biochemical noise is smaller for repressors than for activators. Finally, Equation 21 of the main text is obtained by neglecting the term 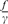 in Equation 53.

If the response times of the enzyme and the transcription factor are not equal, the same analysis gives
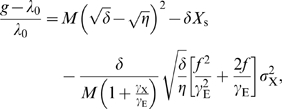
(54)where *γ*_X_ is the degradation rate, i.e., the response time, of the transcription factor and *γ*_E_ is the degradation rate (response time) of the enzyme. This shows that the effect of the fluctuations in the transcription factor concentration, *X*, critically depends upon the response times of *X* and *E*: only when *X* fluctuates more slowly than the time scale on which *E* can respond to these fluctuations (*γ*_X_<*γ*_E_), are the fluctuations in *X* propagated effectively to fluctuations in *E*. In contrast, if the fluctuations in *X* are fast compared to the response time of *E* (*γ*_X_>*γ*_E_), then the slow enzyme dynamics will effectively integrate out the fluctuations in *X*; indeed, the last term on the right hand side of the above equation is then small.
